# Towards Neuro-CoViD-19 

**DOI:** 10.6026/97320630016288

**Published:** 2020-04-30

**Authors:** Francesco Chiappelli

**Affiliations:** 1 UCLA Center for the Health Sciences, Los Angeles, California, USA

**Keywords:** Angiotensin-converting enzyme-2 receptor (ACE2-R), Central Nervous System (CNS), Corona Virus Disease 2019 (CoViD-19), Severe Acute Respiratory Syndrome Corona Virus-2 (SARS-CoV-2), transmembrane protease serine-2 (TMPRSS2)

## Abstract

CoViD-19 is the current pandemic caused by the Severe Acute Respiratory Syndrome Corona Virus-2 (SARS-CoV-2). Infection by SARS-CoV-2 occurs via the binding of its S protein to the angiotensin-converting enzyme-2 receptor (ACE2-R). S binding to ACE2-R leads to a drop in ACE2, a homolog of angiotensin converting enzyme (ACE). In the central nervous system (CNS), ACE mediates neuroinflammation, neurodegeneration and neurotoxicity responsible for several CNS disorders. ACE2 counteracts the damaging effects of ACE on CNS neurons. SARS-CoV-2 can directly access the CNS via the circulation or via cranial nerve I and the olfactory bulb. Inactivation of ACE2 following binding of SARS-CoV-2 S protein to ACE2-R in situ might blunt ACE2-moderating effects upon ACE CNS neurotoxicity and neurodegeneration. Here, we propose a neurobiological mechanism directly involving SARS-CoV-2 binding to ACE2-R in the etiology of putative Neuro-CoViD-19.

## Background

The novel Corona Virus first reported late in 2019 (nCov-19) was recently renamed Severe Acute Respiratory Syndrome Corona Virus-2 (SARS-CoV-2). SARS-CoV-2 causes the Corona Virus Disease 2019 (CoViD-19), designated a global pandemic by the World Health Organization (WHO). SARS-CoV-2 is a positive-sense single stranded RNA (+ssRNA) virus that is highly contagious in human-to-human transmission with a significant morbidity, and a mortality rate estimated on the average of 3.4%, but reaching as high as 6.5-10% in the elderly. To minimize confusion among the diverse SARS corona viruses identified to date, WHO recommends the terminology rather than 'SARS-CoV-2, the virus responsible for CoViD-19" (WHO, 24 Feb. 2020).

As is the case for other members of the coronaviridae family, SARS-CoV-2 consists of four structural proteins: S (spike), E (envelope), M (membrane), and N (nucleocapsid, which contains the RNA genome). S, E, and M form the viral envelope. S allows attachment of the virus to the host cell membrane via the angiotensin-converting enzyme-2 receptor (ACE2-R) [1, 2], following its priming by transmembrane protease-serine 2 (TMPRSS2) [3]. This observation has raised the possibility of using TMPRSS2 inhibitors to prevent S priming, and as potential anti-viral against SARS-CoV-2; but, the physiological role of TMPRSS2 is not fully understood, making the deployment of that strategy hazardous.

SARS-CoV-2 infection leads to severe interstitial pulmonary pathology characterized by diffuse, chronic inflammation of the lungs beyond the terminal bronchioles, fibrosis and collagen formation in the alveolar walls with infiltration of large mononuclear cells in the alveolar spaces. The symptoms of SARS-CoV-2-induced interstitial pneumonia include progressive dyspnea, clubbing of the fingers, cyanosis, and fever eventually leading to acute and lethal lung failure in the more susceptible patients. Patients who recover from CoViD-19 can retain extensive irreversible damage to the lung tissue [4].

ACE2 has a protective effect against SARS-CoV-2-induced lung injury by increasing the production of angiotensin1-7 [5]. However, S binding to ACE2-R leads to a drop in ACE2 [4].

While asymptomatic, patients may still retain a certain level of viremia hidden in tissue reservoirs throughout the body. These pools of inactive SARS-CoV-2 may be ready for re-activation pending appropriate biological stimuli, in a manner akin to the reservoirs of hidden HIV in asymptomatic AIDS patients.

ACE2 is a nearly ubiquitous exopeptidase critical to the metabolic role of the vasodilator-active heptapeptide in the renin-angiotensin system, angiotensin1-7. ACE2 mRNA is present in virtually every organ, including oral and nasal mucosa, nasopharynx, lung, gastro-intestinal tract, lymphoid tissues, renal and urinary tract, reproductive organs, as well as the brain [6]. Its protein expression in the nasal mucosa, the epithelia of the nasopharyngeal, pulmonary and gastro-intestinal systems may provide some of the major routes of entry for SARS-CoV-2. Ports of entry of SARS-CoV-2 in to the central nervous system (CNS) may include the arterial circulation of the posterior carotid artery or the circle of Willis when viremia is notable, or directly through the endothelium of the vasculature intimately associated with cranial nerve I and the olfactory bulb at the time of initial infection.

The renin-angiotensin regulatory loop, renin–angiotensin system, or renin-angiotensin-aldosterone system, is a hormone system that regulates blood pressure and fluid and electrolyte balance, as well as systemic vascular resistance, and as such modulates the cardiovascular system, including the heart and blood vessels, and its physiological balance with the pulmonary and the renal systems. The renin-angiotensin system also modulates certain CNS activities, including neuroprotection, cognitive and meta-cognitive function, and physiologic regulation of the cholinergic, dopaminergic and adrenergic systems [7]. Angiotensin1-7 has anti-oxidant and anti-inflammatory effects, which are cytoprotective by increasing the expression of endothelial and neuronal nitric oxide synthase enzymes leading to augmented production of nitric oxide [8,9].

ACE2 is a homolog of angiotensin converting enzyme (ACE). CNS ACE mediates increased activation of oxidative stress, apoptosis and neuroinflammation causing neurodegeneration in several brain disorders. ACE2, by contrast, counteracts the damaging effects of ACE on CNS neurons [9,10]. ACE2-R is expressed by glial and neuronal CNS cell populations [11]. Taken together, the data suggest that it is possible and even probable that inactivation of ACE2 following binding of SARS-CoV-2 S protein to ACE2-R in situ blunt ACE2-moderating effects upon ACE CNS neurotoxicity and neurodegeneration.

In brief, the evidence to date proffers a neurobiological mechanism directly involving SARS-CoV-2 binding to ACE2-R in the neuropathogenesis, and associated cascade of CNS involvement, from heightened anxiety to blunted cognition, parkinsonian movement disorders and other neuropathies observed in certain cases of CoViD-19, as well neuroanatomical pathology in the CNS endothelium in CoViD-19 post mortem. [11].

The ACE family has wide allelic polymorphisms, including an insertion/deletion (I/D) polymorphism that accounts for over half the variance in plasma ACE level. I/D alleles result in substantial inter-individual genetic variability for high risk of ischemic heart disease and for predicting the efficacy of the antihypertensive therapy [12,13].

The D allele, is distributed in 55-60% of the world population in a characteristic global distribution [13] that remarkably mimics that of the reported natural history of the CoViD-19 pandemic to date [WHO data]: that is, roughly between the 40th and the 45th parallel north. The D allelle is associated with increased activity of the ACE enzyme and is predicted to be associated with pathologies involving increased activity of the renin-angiotensin system both peripherally and in the CNS, including increased risk of hypertension, heart failure, cerebral infarct, diabetic nephropathy, encephalopathy, asthma and pulmonary insufficiency. It follows that the D allele of the ACE family may be associated with the more critical manifestations of CoViD-19 that require hospitalization, ICU and that are eventually lethal, as well as putative Neuro-CoViD-19. By contrast, the I allele seem to be protective of aerobic capacity and lung function in 40-45% of the population [13]. There also appears to be no direct evidence in support of SARS-CoV-2 S-protein binding-resistant ACE2 mutants among different sub-populations of CoViD-19 patients [14].

## Conclusions:

In conclusion, the evidence to date strongly suggests CNS involvement of SARS-CoV-2 infection in certain CoViD-19 patients possibly resulting in a neuropathic form of CoViD-19. Neurobiological findings proffer a mechanism directly involving SARS-CoV-2 binding to ACE2-R in the etiology of putative Neuro-CoViD-19. It is possible and even probable that not every CoViD-19 patients will develop Neuro-CoViD-19, although they all may be at risk via arterial SARS-CoV-2 viremic irrigation of the CNS, and directly via the the vasculature running with first cranial nerve and the olfactory bulb. Future research should establish whether risk of developing CoViD-19 and Neuro-CoViD-19 is related to the expression of the ACE D allele, in addition to age, pre-existing conditions and gender.
